# Multicentre evaluations of two new rapid IgG4 tests (WB rapid and panLF rapid) for detection of lymphatic filariasis

**DOI:** 10.1186/1475-2883-6-9

**Published:** 2007-10-26

**Authors:** Rahmah Noordin, Makoto Itoh, Eisaku Kimura, Rohana Abdul Rahman, Balachandran Ravindran, Rohela Mahmud, Taniawati Supali, Mirani Weerasooriya

**Affiliations:** 1 Institute for Research in Molecular Medicine, Universti Sains Malaysia (USM), Malaysia; 2 Department of Parasitology, Aichi Medical University, Nagakute, Aichi-ken, Japan; 3 Immunobiology Laboratory, Institute of Life Sciences, Bhubaneswar, India; 4 Department of Parasitology, Faculty of Medicine, University of Malaya, Malaysia; 5 Department of Parasitology, Faculty of Medicine, University of Indonesia, Indonesia; 6 Department of Parasitology, Faculty of Medicine, University of Ruhuna, Sri Lanka

## Abstract

In the global effort to eliminate lymphatic filariasis (LF), rapid field-applicable tests are useful tools that will allow on-site testing to be performed in remote places and the results to be obtained rapidly. Exclusive reliance on the few existing tests may jeopardize the progress of the LF elimination program, thus the introduction of other rapid tests would be useful to address this issue. Two new rapid immunochromatographic IgG4 cassette tests have been produced, namely WB rapid and panLF rapid, for detection of bancroftian filariasis and all three species of lymphatic filaria respectively. WB rapid was developed using *Bm*SXP recombinant antigen, while PanLF rapid was developed using *Bm*R1 and *Bm*SXP recombinant antigens. A total of 165 WB rapid and 276 panLF rapid tests respectively were evaluated at USM and the rest were couriered to another university in Malaysia (98 WB rapid, 129 panLF rapid) and to universities in Indonesia (56 WB rapid, 62 panLF rapid), Japan (152 of each test) and India (18 of each test) where each of the tests underwent independent evaluations in a blinded manner. The average sensitivities of WB rapid and panLF rapid were found to be 97.6% (94%–100%) and 96.5% (94%–100%) respectively; while their average specificities were both 99.6% (99%–100%). Thus this study demonstrated that both the IgG4 rapid tests were highly sensitive and specific, and would be useful additional tests to facilitate the global drive to eliminate this disease.

## Findings

Diagnostic tools are an essential component for the success of the Global Program for Elimination of Lymphatic Filariasis (GPELF). Thus far, the established diagnostic tests that are commercially available for bancroftian filariasis are two antigen detection tests namely NOW Filariasis Test [1] and Og4C3-ELISA (Trop Bio, Pty. Ltd., Australia); and for brugian filariasis is the Brugia Rapid test [2]. A laboratory-based Bm14-ELISA has also been extensively employed in studies in Egypt [3,4]. In addition PCR-based assays for both brugian and bancroftian filariasis are also promising tools for the GPELF which can be employed for monitoring infections in both human and vector [5,6]. LF mainly affects the poor who reside in areas which are remote and/or without adequate health and laboratory facilities. Thus diagnostic tools in the format of rapid tests, particularly those based on immunochromatography technology, are most suitable to be employed for the GPELF, since they allow easy on-site testing, followed by rapid, simple reading and interpretation of results. These would avoid potential logistical challenges for sample storage and transportation, as well as more serious problems such as sample mix-up due to unclear/unreadable labels and sample degradation that may occur if collection and performance of tests are not conducted at the same or nearby locations. For such a major global program which needs to be sustained for a prolonged period, availability of a panel of rapid tests would help ensure smooth progress of the program and avoid potential problems such as supply interruption and changes/variations in test performance. Two new rapid immunochromatographic cassette tests based on detection of anti-filarial IgG4 antibody are now commercially available namely WB rapid and panLF rapid. The aim of this study is to perform a multicentre study to validate the sensitivities and specificities of the tests.

The test kits were acquired by the senior author from the manufacturer. A proportion of the tests were validated at USM, and the rest of the tests were couriered to the other four participating laboratories. The WB rapid test consists of two lines namely a test line and a control line, with the former comprising *Bm*SXP recombinant antigen.

The panLF rapid test consists of three lines namely two test lines, one comprising *Bm*SXP and the other *Bm*R1 recombinant antigens; and a control line. Goat anti-mouse IgG antibody is employed as the control line for both tests. These lines are invisible in an unused test and are coloured red after performance of the test. Serum/plasma and whole blood may be employed as test samples.

For serum samples, the test is performed by delivering 25 ul serum sample into the square bottom well. When the sample front reaches the blue line on the cassette window, two drops of buffer are added to a top oval well to release the conjugate solution (monoclonal anti-human IgG4 conjugated to colloidal gold). This is followed by pulling a plastic tab at the bottom of the cassette and adding a drop of buffer into the square bottom well, and by 15 minutes, the results can be read. For both tests, appearance of only the red control line denotes a negative result. For WB rapid test, a positive result is demonstrated when two red lines (a test and a control line) are seen. For panLF rapid test, a test is interpreted as positive when either three red lines (two test lines and a control line) or two red lines (a test and a control line) are observed.

Each participating institutions employed serum samples from their serum bank, which were obtained according to the ethical requirements of the respective organizations. With regard to the samples tested in Japan, the sera from *W. bancrofti *patients were collected in Sri Lanka, while the normal sera were from Japanese. The tests were performed in a blinded manner and the results were collected from each centre by e-mail attachments.

Table 1 shows the number of the tests and the results obtained at each institution. WB rapid test displayed an average sensitivity of 97.6% (239/245), ranging from 94% to 100%. The average overall sensitivity of panLF rapid test was 96.5% (390/404), ranging from 94% to 100%; the sensitivity for detection of *W. bancrofti *infection was 96.0% (217/226) [94% to 100%] while that for detection of brugian filariasis was 97.2% (173/178) [92% to 100%]. The specificities of both tests were evaluated with serum samples from quite a large variety of other infections, which included helminthes, protozoan, bacterial and viral infections. The results showed that the tests were either 99% or 100% specific, with an average specificity of 99.6%. Thus, at all the evaluation centres, the sensitivites and specificities of both tests were consistently high.

**Table 1:** Sensitivities and specificities of WB rapid and panLF rapid tests evaluated at five institutions.

	WB rapid					**panLF rapid**				
	Wb	Bm/Bt	*Other infec-tions	Nor-mals	Sens & Spec	**Wb**	**Bm/Bt**	*Other infections	**Normals**	**Sens & Spec**

UI	43 (43)	-	0 (13)	-	100% sens 100% spec	**24 (24)**	**35 (38)**	**-**	**-**	**Overall** **sens: 95%** **Wb: 100% sens** **Bm: 92% sens**
UM	30 (30)	-	0 (58)	0 (10)	100% sens 100% spec	**30 (30)**	**29 (29)**	**0 (60)**	**0 (10)**	**100% sens (Bm & Wb)** **100% spec**
AMU	102 (104)	-	-	0 (48)	98% sens 100% spec	**99 (104)**	**-**	**-**	**0 (48)**	**95% sens (Wb)** **100% spec**
USM	47 (50)	-	1 (65)	0 (50)	94% sens 99% spec	**47 (50)**	**109 (111)**	**1 (65)**	**0 (50)**	**Overall sens: 97%** **Wb: 94% sens** **Bm: 98% sens** **99% spec**
ILS	17 (18)	-	-	-	94% sens	**17 (18)**	**-**	**-**	**-**	**94% sens (Wb)**

**Note**: Numbers indicate number of samples that were positive, and numbers in parenthesis were the number of samples tested.

UI : University of Indonesia, Indonesia

UM : University of Malaya, Malaysia

AMU : Aichii Medical University, Japan

USM : Universiti Sains Malaysia, Malaysia

ILS : Institute of Life Sciences, India

sens : sensitivity; spec : specificity

Wb: *Wuchereria bancrofti *; Bm: *Brugia malayi *; Bt: *Brugia timori*

Filarial samples tested in Indonesia, Malaysia and India came from mf+ individuals; while those tested in Japan also came from individuals who are mf-, CFA+ (>512 units).

*Other infections: ascariasis, trichuriasis, hookworm, strongyloidiasis, toxocariasis, toxoplasmosis, typhoid, cysticercosis, schistosomiasis, malaria, dengue, amoebiasis

WB rapid: sensitivity : 97.6% (239/245); specificity : 99.6% (243/244)

panLF rapid: overall sensitivity : 96.5% (390/404); sensitivity for Wb detection: 96.0% (217/226); sensitivity for Bm/Bt detection: 97.2% (173/178); specificity: 99.6% (232/233)

The mf+ samples with circulating filarial antigen (CFA), as determined by Og4C3 assay in samples from Sri Lanka (n = 41) and India (n = 18), had CFA values > 512 and >1000 respectively. The Sri Lankan mf- samples had CFA values > 512; with 62/63 (98.4%) and 59/63 (96.7%) samples positive for WB rapid and panLF rapid respectively. In addition, the two rapid tests were also tested with samples from 22 amicrofilaraemic, CFA+ individuals (cryptic infections) from India which had a wider range of CFA values (100–4786). In general both rapid tests tested positive with sera which had CFA units greater than 200, and they tested negative with sera which had CFA units below this value. Since CFA may remain positive for sometime after death of adult worms, some of the mf-, low CFA+ individuals may no longer be actively infected. On the other hand these may also be individuals with reproductively immature worms.

In the pre-certification phase of the elimination program and in the surveillance activities post-elimination, a highly sensitive test, as displayed by an antibody-based diagnostic tool is essential since the level of infection, if any, is very low. Therefore, although a rapid antigen detection test is already available for bancroftian filariasis, an antibody detection assay would probably be more useful in the screening of young children as required in the pre-certification phase of GPELF. Antigen detection assays depend on presence of developmentally mature worms while antibody assays could potentially detect exposure to infective larvae by children. Thus WB rapid would be helpful to address this diagnostic requirement. For detection of all species of lymphatic filariasis, a rapid test such as panLF rapid would be very useful in several kinds of situations, namely testing in areas where there are mixed bancroftian and brugian filaria infections, in areas where the infecting species is not known or not confirmed, and for screening of immigrant workers in countries such as Malaysia which has more than 1.3 million workers from filarial endemic countries. These workers may pose a threat to the achievement of the disease elimination or they may be a source of resurgence of the disease in the future.

*Bm*R1 is a recombinant antigen derived from *Bm17DIII *gene [GenBank: AF225296] and employed in a rapid test called Brugia Rapid. It has been shown to be highly sensitive (>95%) and specific (≥ 99%) for detection of *B. malayi *and *B. timori *infections in laboratory evaluations [7-10] and field studies [11-14]. In a field study in Malaysia which is a low endemic area, Brugia Rapid detected about ten times more positive cases than parasitological diagnosis, while in the high endemic area of Indonesia, the increase in detection was about three times [11,12]. Follow-up post-treatment studies of mirofilaraemic individuals showed that the titres of IgG4 antibodies to *Bm*R1 decreased post-treatment. In Malaysia which is a low endemic area, it took approximately 6 months to 2 years post-treatment for the assay to become negative [15,16]. In a study involving a paediatric population in Kerala, ultrasonography ('filarial dance sign' or FDS) identified adult worms in 7 out of the 39 (18%) amicrofilaraemic children who were Brugia Rapid positive, thus providing definitive evidence that the rapid test detected active infection. This was comparable to the observation of FDS in 6 out of 32 (19%) microfilaraemic children [17].

*Bm*SXP is a recombinant antigen derived from *SXP1 *gene [GenBank no: M98813], the clone was isolated from a *B. malayi *adult male worm cDNA library with sera of bancroftian filariasis patients [18]. A rapid flow-through IgG immunofiltration test using *Wb*SXP recombinant antigen has been developed and a sensitivity of 91% (30/33) was recorded for detection of *W. bancrofti *infection [9].

In a recent study, *Bm*SXP was found to be more sensitive (95%) in detecting *W. bancrofti *infection as compared to *Bm*R1 (14%). On the other hand *Bm*R1 was more sensitive than *Bm*SXP in detecting *B. malayi *infection (98% and 84% respectively) [19]. Since *Bm*R1 and *Bm*SXP recombinant antigen cross-reacts with bancroftian and brugian filaria infection sera respectively, the panLF rapid test is not useful for species identification. However in the context of GPELF or for screening of foreign workers, this does not pose a problem. Cross-reactivities with *Loa-loa *and *Onchocerca *infection sera were observed with both rapid tests, thus they are not useful in areas co-endemic with these infections. However the tests may be employed in the vast lymphatic filariasis endemic areas in the world, particularly in Asia, which do not overlap with endemic areas for non-lymphatic filariasis. Since LF endemic areas are also prevalent for infections with soil-transmitted helminthes and intestinal protozoa, the high specificities shown by both rapid tests with respect to non-filarial infections would allow the tests to be used with high confidence in these areas.

In conclusion, the present multicentre evaluation study conducted in five institutions (located in four different countries) clearly demonstrated the high sensitivities and specificities of WB rapid and panLF rapid tests. Thus these tests should be employed in further field studies and would merit consideration as potential tools to assist in the GPELF.

## Competing interests

RN, with the assistance of RAR, developed WB rapid and panLF rapid tests

## Authors' contributions

RN – conceive, design and supervise the study, participated in the evaluation at USM, analysed the results, wrote the first draft of the manuscript.

RAR – performed the evaluation and participated in the analysis of the results at USM.

IM & KE-supervised and participated in the evaluation at Aichi Medical University, edited the manuscript.

RB – supervised and participated in the evaluation at the Institute of Life Sciences, edited the manuscript.

RM – supervised and participated in the evaluation at University of Malaya, edited the manuscript.

ST – participated in the evaluation at University of Indonesia, edited the manuscript.

WMV – supervised and participated in the serum sample collection in Sri Lanka, edited the manuscript.

All authors read and approved the final manuscript
